# A Public Value Crisis Model Approach to COVID-19 Outbreak Control in the Dominican Republic

**DOI:** 10.7759/cureus.98766

**Published:** 2025-12-08

**Authors:** Amado A Baez, Robert Paulino, Carlos Ruiz-Matuk, Ingrid Ruiz, Erick Sanchez

**Affiliations:** 1 Research, Universidad Nacional Pedro Henríquez Ureña, Santo Domingo, DOM; 2 Emergency Medicine, Augusta University Medical College of Georgia, Augusta, USA; 3 Tropical Medicine Institute, La Universidad Iberoamericana (UNIBE), Santo Domingo, DOM

**Keywords:** covid-19, dominican republic, health policy making, outbreak control, public value crisis model

## Abstract

Background: COVID-19 exposed critical vulnerabilities in health systems worldwide. The Dominican Republic faced an early and severe outbreak in Duarte Province, necessitating rapid action. The government implemented a localized “Public Value Crisis Model,” known as #PlanDuarte, integrating public-private collaboration, epidemiological intelligence, and community engagement.

Objective: To evaluate whether #PlanDuarte was associated with measurable epidemiological improvements using Interrupted Time Series Analysis (ITSA), and to examine how public value principles informed crisis governance.

Methods: We analyzed daily COVID-19 data from March to December 2020 using segmented regression with Newey-West standard errors. Duarte Province (intervention site) was compared with all other provinces (non-intervention group). Data sources included the Ministry of Public Health registry. We assessed changes in trends for daily new cases following the lockdown and the implementation of public value-driven actions.

Results: Prior to the intervention, Duarte experienced a significant daily increase in cases (β1 = 0.88, p < 0.001). Following implementation, the post-intervention trend decreased significantly (β3 = -1.63, p < 0.001), unlike the non-intervention provinces. Epidemiological improvements corresponded with increased testing, expanded hospital capacity, and strengthened intersectoral coordination.

Conclusion: The intervention in Duarte Province was associated with reduced epidemic growth and strengthened system responsiveness. Rooted in public value theory, #PlanDuarte illustrates how coordinated, community-empowering governance structures can improve crisis outcomes. Results reflect temporal association rather than definitive causality and underscore the need for context-sensitive governance in middle-income settings.

## Introduction

Emergence of severe acute respiratory syndrome coronavirus 2 (SARS-CoV-2) following a cluster of unexplained pneumonia in the city of Wuhan, China, in the late months of 2019 [1] provoked an unprecedented global health impact. Later, on January 30, 2020, the World Health Organization (WHO) declared the outbreak a public health emergency of international concern (PHEIC) and produced the first guidelines for country preparation and response plans, focused on detection, preparation, and rapid response to the outbreak [2]. These guidelines, along with the first sequences and laboratory assays, were delivered to all regional offices [2,3].

COVID-19 posed an unprecedented public health challenge for the Dominican Republic (DR), exposing gaps in surveillance, hospital capacity, interagency coordination, and community engagement. Duarte Province quickly became the country’s earliest epicenter, prompting the implementation of #PlanDuarte, a targeted “Public Value Crisis Model” intervention designed to integrate epidemiological intelligence, public-private partnerships, and community mobilization.

Meanwhile, in the DR, the first official case was registered on February 29, 2020, in a European visitor located in the National District; subsequently, a second case was confirmed in another European visitor in Duarte Province, in Villa Riva locality in the north-central plateau. Both cases were asymptomatic at the moment of identification, and both survived the infection [3].

Evidence suggests that the ancestral SARS-CoV-2 lineage exhibited broader and more sustained transmission potential than SARS-CoV-1, with effective reproductive numbers (Rt) that were consistently higher and less prone to abrupt interruption. In practical terms, SARS-CoV-2 transmission chains were less likely to terminate early, enabling more reliable and sustained spread compared with the dynamics observed during the SARS-CoV-1 outbreak [4,5]. In Europe, the 614G variant was first observed in genomes sampled on January 28 in a small outbreak in Bavaria, Germany, which was initiated by a visitor from Shanghai and subsequently controlled through public health efforts. It is therefore likely that the D614G mutation occurred in China before being introduced on multiple occasions to European countries, where it increased in frequency. This scenario is consistent with the rapid increase in February and March of European virus genomes that carried the 614G variant.

COVID-19 created unprecedented challenges for health systems globally. The DR detected its first cases in March 2020 but rapidly experienced a concentrated surge in Duarte Province, which became the national epicenter early in the pandemic. This required a decisive and coordinated response.

On March 31, 2020, the president of the DR, via Decree 140-20, created the COVID-19 Emergency Committee. This Presidential Committee was tasked with creating public-private partnerships as well as developing public policy, strategies, and operations to combat COVID-19 at a national level. The committee presented on April 5 a comprehensive technology utilization, hospital capacity augmentation, and Test-Trace-Treat strategy with a focus on strengthening local government capacities via public-private partnerships. At the heart of the committee was the engagement of all possible public and private actors, creating synergies and scale that would result in strong public value [6-10].

Even though the country was not fully prepared to respond to a public health catastrophe of this magnitude, it was able to avoid worse outcomes by taking bold and decisive actions to minimize COVID-19 transmission. Testing strategies served as a critical tool for identifying the extent of viral spread within the community at any given time, while equal importance was placed on monitoring hospital bed availability, maintaining outbreak situational awareness, communicating effectively with the public, and coordinating field response activities across the relevant organizations [11].

To address the crisis, the Dominican government deployed a targeted intervention, #PlanDuarte, rooted in a Public Value Crisis Model to integrate public health measures, community mobilization, and intersectoral coordination. Guided by epidemiological intelligence from the newly created C5i Epidemiology Intelligence Center, the intervention focused on flattening the curve, expanding hospital capacity, and reducing community transmission.

Study objectives

The study aims to evaluate the implementation of the Public Value Crisis Model (#PlanDuarte) during the COVID-19 outbreak in Duarte Province; quantify the temporal association between the intervention and changes in COVID-19 epidemiological indicators using Interrupted Time Series Analysis (ITSA); analyze public value components, community engagement, interagency coordination, and public-private partnerships and their role in shaping outcomes; and provide a conceptual and empirical framework for crisis governance in middle-income countries.

This article was previously posted to *The Lancet* preprint server on October 19, 2021.

## Materials and methods

Pillars of rapid response

Rapid response during a pandemic relies on four conceptual pillars: (1) Testing Capacity, identifying, confirming, and isolating cases early; (2) Contact Tracing, locating exposed individuals to prevent further transmission; (3) Treatment and Hospital Capacity, ensuring sufficient ICU beds, ventilators, trained staff, and emergency command systems; and (4) Risk Communication and Community Engagement, creating trust, promoting adherence, and empowering communities.

These pillars are universally recognized but manifest differently based on a country’s resources, infrastructure, and institutional maturity. In the DR, these pillars were adapted to local realities, informing the development of #PlanDuarte.

Although global examples such as Singapore and South Korea demonstrated the effectiveness of strong governance and rapid response, we acknowledge that these high-income countries possess significantly greater technological infrastructure, prior outbreak experience such as severe acute respiratory syndrome (SARS) and Middle East respiratory syndrome (MERS), and digital capabilities. Our study references them as conceptual benchmarks, not comparators. The first line of defense from the Singapore health officials was to reduce the importing of cases from Wuhan by screening temperature levels at ports, following up patients at home, providing masks to asymptomatic patients and their family members, and extending screening measures to the community level, making screening one of the pillars of a rapid response [12].

Massive testing within the context of a global pandemic should be the quintessential tool to stop the fast spreading of a highly infectious disease. Massive testing, while costly, reduces the economic impact of hospitalizations and resources that might be used for treating patients. The real-time polymerase chain reaction (RT-PCR) is the gold standard for diagnosis, and at times, shortages have been reported due to the high demand, a consequence of the quickly spreading COVID-19. The Singapore healthcare officials tried to work on their experience with the 2002 SARS outbreak by working on rapidly available biotechnology and personnel to operate that technology in the case of a future pandemic [12].

Contact tracing quickly became a trend to follow up asymptomatic patients, who comprise a large pool of cases that can also spread the disease, with reports stating that as much as 40 to 45% of patients do not present symptoms at any stage. The biggest difference with previous pandemics is that this is the digital era, where countries such as South Korea have used technology to spread useful information, encouraging hand washing, the usage of masks, and social distancing, efficient testing and analysis, and the usage of geotagging for tracing asymptomatic patients [13,14].

If anything, the COVID-19 pandemic has accentuated the socioeconomic differences between the upper and working classes, as it has affected tourism, education, professional sports, and even healthcare. The new normality forced the rapid expansion of telehealth, which is poorly accessible to those in the working classes of low-income countries because of a lack of access to the internet.

With the introduction of the new normality, a new term arose, namely, a national lockdown. Travel of people for essential activities and contact is the cornerstone of COVID-19. Encouraging social distancing by shutting down countries is the most effective way to stop transmission. Having a lockdown slows down community transmission, aids in the isolation of asymptomatic patients and contacts, and helps quarantine. In Wuhan, the introduction of the lockdown helped massively in the containment, mitigation, and suppression when there was neither an effective drug nor vaccine, taking measures that included traffic restriction, prohibition of social gatherings, and home quarantine that proved to be highly effective in controlling the quick spread of COVID-19 within a city with 10 million residents [15].

The positive impact of technology within the context of a global pandemic equally has a negative counterpart. Misinformation spreads as fast as useful information, whether it is from official or unofficial sources. There have been reports from Latin American countries such as the DR, where initial efforts to slow down the rate of infection were seen as an overreaction from the government, with concerns especially regarding the economic impact a national lockdown would have on daily life [16].

**Figure 1:**
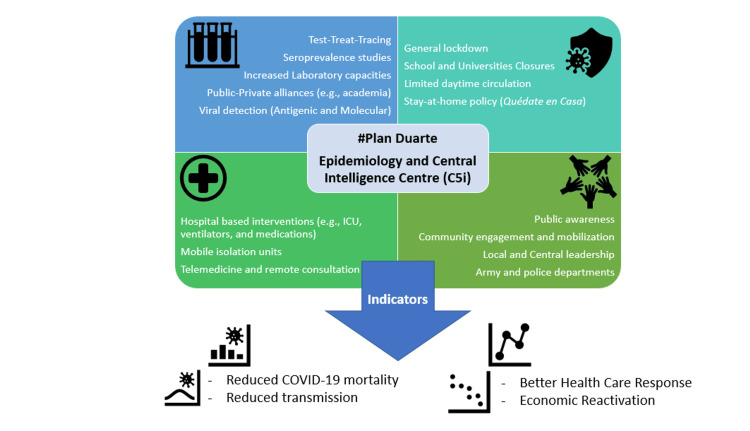
#PlanDuarte Public Value Model This figure is an original creation of the authors

The centerpiece of the strategy for the DR COVID-19 Emergency Committee was the "Public Value Crisis Model" piloted on April 12, 2020, at San Francisco de Macoris (SFM), Duarte Province. #PlanDuarte [11] targeted SFM as the highest case and mortality city at that time (Figure 1). Within two weeks of implementing #PlanDuarte the province started showing evidence of a positive impact in reported positivity, critical care use, and mortality [12]. In early May, the focused and targeted approach to Duarte Province resulted in a statistically significant impact on the Case Fatality Ratio for the whole country [13,14]. The Public Value in Crisis Model was reproduced successfully in multiple other provinces and, more recently, became the hallmark of the National District strategy under the leadership of Mayor Carolina Mejia. On April 22, 2020, the first Dominican Epidemiology Intelligence Center was developed by the Presidential COVID-19 Committee under the leadership of Minister of Defense Lt General Paulino Sem, Counter Admiral Lee Ballester, and Dr. Amado Alejandro Baez. The Epi Intel Center was housed out of the Ministry of Defense C5i Center [15] and tasked with creating interagency "Intelligence Fusion" efforts that guided country-wide strategies and COVID-19 operations [16]. After the successful implementation of the C5i Epidemiology Intelligence Fusion Center, the Dominican Ministry of Public Health viewed this model as especially important and effective and proceeded to reproduce this effort four months later in August 2020, creating the MOH "Center for Public Health Intelligence" [17,18].

Public value model

Public value is value for the public. It equates managerial success in the public sector with initiating and reshaping public sector enterprises in ways that increase their value to the public in both the short and the long run. Public value describes the value that an organization contributes to society. The term was originally coined by Harvard professor Mark H. Moore, who saw it as the equivalent of shareholder value in public management. Public value is supposed to provide managers with a notion of how entrepreneurial activity can contribute to the common good. Management concepts such as shareholder value, stakeholder value, customer value, sustainability, or corporate social responsibility should legitimize themselves regarding their impact on the common good [17]. In his social psychological-based concept, public value emerges for individuals from the experiences made in social structures and relationships [18].

Public value theory, as defined by Mark Moore, asserts that government legitimacy arises from the creation of outcomes valued by society, delivered through coordinated public action, and supported by an authorizing environment.

The Public Value Crisis Model implemented in #PlanDuarte included a clear articulation of value such as flattening the curve, reducing mortality, and expanding capacity; performance measurement through CFR, positivity rate, and hospital bed and ICU availability; external accountability involving engagement with local authorities, business leaders, and the press; and distributed internal accountability through use of the Incident Command System (ICS) with integrated military, police, public health, and municipal leadership. These elements align with public value’s strategic triangle of valued outcomes, operational capacity, and authorizing environment.

In the context of COVID-19, #PlanDuarte operationalized this model through internationally accepted epidemiological benchmarks such as flattening the curve, reducing positivity, expanding hospital capacity, and lowering CFR. The model emphasized public-private collaboration, community empowerment, and intelligence-driven decision-making, forming an integrated crisis-response framework.

**Figure 2:**
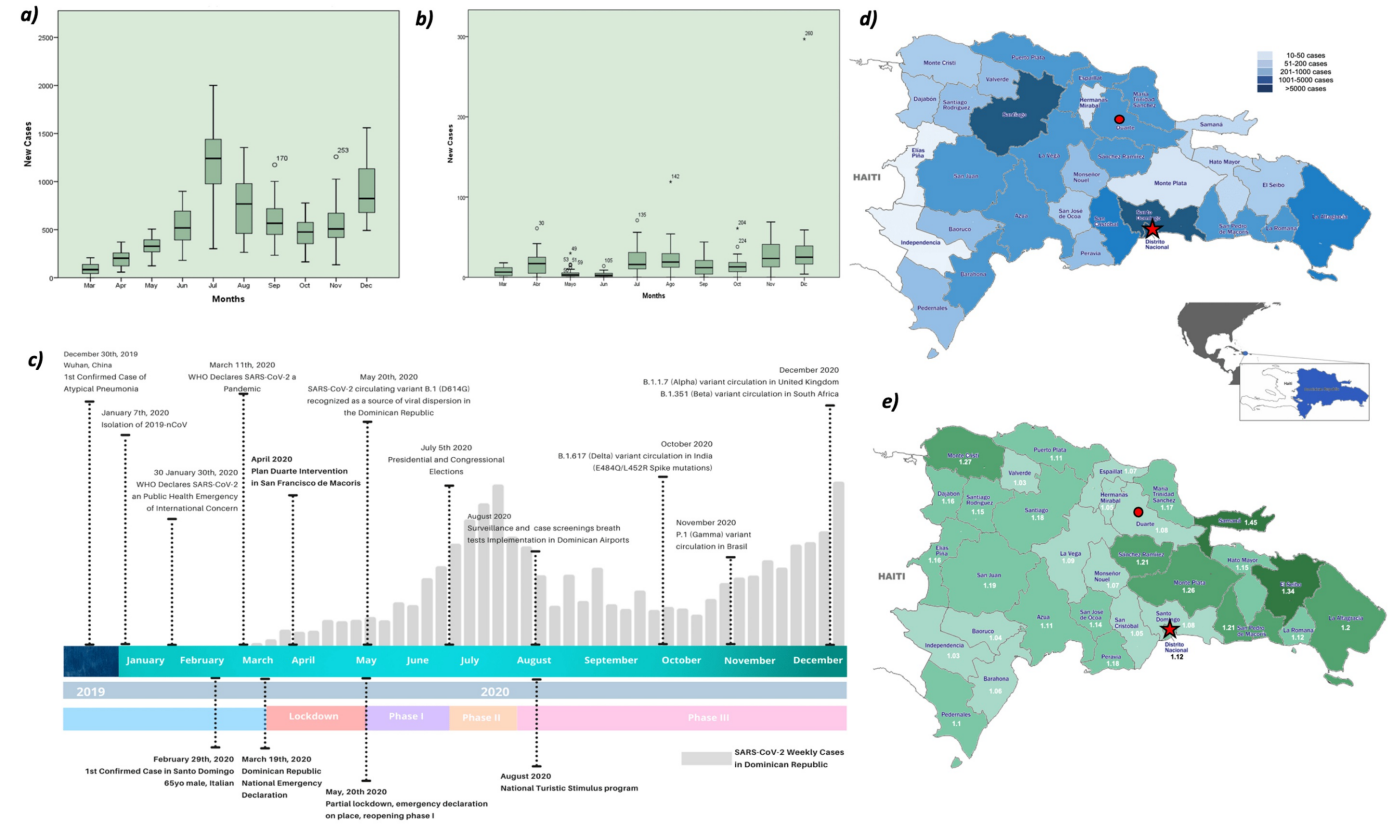
(a) Boxplot analysis of SARS-CoV-2 reported cases in quartiles from March to December 2020 in the Dominican Republic (all provinces); (b) reported cases in Duarte's province only; (c) timeline of relevant epidemiological dynamics in the DR; (d) frequencies of reported cases in each province; e) effective reproductive number (Rt) of COVID-19 cases per provinces. Duarte's province is marked with a green circle, and the capital city Santo Domingo depicted with a red star. This figure is an original creation of the authors.

As of April 4, 2020, a total of 5926 cases were confirmed in the DR, 9.3% from Duarte Province, and a positivity rate of 37.6% (n = 562), an incidence rate 188 times higher compared with the National District, the capital city, which had a positivity rate of 22.1%. Accumulated deaths in the capital city were 37 cases versus 75 in Duarte (Figure 2), representing twice the number seen in the most populous city in the country.

Timeline of interventions

The timeline of interventions included the period from March 1 to 20, 2020, which showed a rapid case increase in Duarte and surveillance constraints. On March 24, 2020, #PlanDuarte was launched as a localized pilot intervention that included a province-wide lockdown, expansion of testing and community surveillance, doubling of hospital capacity that encompassed beds, ICU units, and ventilators, deployment of home-visit teams and isolation units, and creation of the epidemiological intelligence fusion structure C5i. On April 5, 2020, a national strategy was released that adopted several elements pioneered in Duarte.

Community intervention

Strategic elements of Plan Duarte included (1) a province-wide lockdown policy fully enforced by police and military assets beginning on March 24, 2020; (2) enhancement of testing capacity through community surveillance with antibody kits and optimization of PCR testing capacity; (3) enhancement of the hospital system, both public and private, doubling hospital capacity to 77 COVID beds, 23 COVID ICU units, and 21 ventilators; (4) root-cause analysis of crowding clusters with key interventions at banks and markets; (5) implementation of the Incident Command System with the military, police, municipal political leadership, and the public and private health systems along with community leaders; (6) decompression of hospital utilization through deployment of 15 medicalized home-visit units, a COVID-19 hotline, and 199 COVID-19 isolation beds for mild and asymptomatic positive patients who could not safely isolate at home; and (7) weekly videoconference consults with community and local press leaders to create ownership and empowerment and to measure effectiveness at the grassroots level.

Public value standard output

The Public Value Standard output involved articulating a clear and compelling idea of the public value that the agency existed to produce, expressed through a comprehensive municipal plan with public-private partnerships. It included developing measures to record the agency’s performance in producing that public value using standard COVID-19 indicators such as Case Fatality Ratio, Positivity Rate, and availability of hospital resources. It also required inviting and embracing external accountability for defining and creating that value through active community engagement with business leaders and the press. Finally, it called for creating management systems that distributed internal accountability, with the Incident Command System integrated and adapted with the Public Value elements, socialized and well communicated, and supported by telemedicine and home health visits to decongest the hospital system.

Statistical analysis

To understand the impact of interventions, we used ITSA based on segmented regression models to evaluate the impact of a lockdown policy as the public health intervention in Duarte Province on the trends of COVID-19 cases, compared with all provinces except Duarte, where the lockdown and interventions were not performed. These models were fitted utilizing segmented longitudinal data.

The Newey-West method was selected, and several diagnostic and sensitivity assessments were performed. The results of the Dickey-Fuller statistics showed that the residuals were stationary and normally distributed (Z = -5.821, p = 0.000 for Duarte cases and Z = -3.638, p = 0.005 for all cases except Duarte). Ordinary least squares regression models with a time series specification, including an intercept and a trend term, a level, and a trend change, were used to verify serially correlated errors by visually inspecting the residuals and plotting the autocorrelation and partial autocorrelation functions. The Durbin-Watson test was also utilized to assess autocorrelation between data in the generalized least squares regression models for Duarte’s new cases (χ^2^ = 0.306, p = 0.580) and for all provinces except Duarte’s new cases (χ^2^ = 1.267, p = 0.260).

The following segmented regression model was built:

$Y= &beta;0 + &beta;1\times \text{time}_{t} + &beta;2\times \text{Intervention}_{t} + &beta;3\times \text{After Intervention}_{t}$

β0 is the initial number of new confirmed cases at the start of the study. Time is the temporal interval from baseline. β1 is the slope of the number of new confirmed cases before the implementation of the public health policy. β2 and β3 are the slopes of the numbers of new confirmed cases on the first day of the policy implementation and in the following days, respectively. After Policy is the time passed after the policy implementation. Results were calculated with their 95% confidence interval (CI), and p-values set at values <0.05 were considered significant. All statistical analyses were done by means of Stata V15; all scales and scoring systems used are open source and free to use.

Data sources and study period

Epidemiological data were obtained from the official Dominican Republic Ministry of Public Health surveillance registry SIVIGILA (Sistema Nacional de Vigilancia en Salud Pública). The dataset includes daily counts of confirmed COVID-19 cases, tests performed, positivity rate, hospital bed occupancy, ICU occupancy, ventilator availability, and mortality. The analysis covers the period March 1 to December 31, 2020.

Operational definitions

Positivity rate was defined as PCR-confirmed cases divided by total PCR tests multiplied by 100. The case fatality ratio (CFR) was defined as total deaths divided by total confirmed cases multiplied by 100. Hospital capacity referred to the number of available COVID-19 beds, ICUs, and ventilators. Daily new cases represented the newly reported PCR-positive cases.

Model specification and analytical approach

ITSA was used to evaluate the temporal association between the implementation of #PlanDuarte and changes in the trajectory of COVID-19 cases. A segmented regression model was applied, incorporating Newey-West standard errors to account for autocorrelation in the time series data. The model took the form Y = β0 + β1 (time) + β2 (intervention) + β3 (time after intervention), allowing estimation of both level and slope changes following the intervention. The intervention point was set at March 24, 2020, corresponding to the formal implementation of the lockdown and related public value actions in Duarte Province.

To ensure the robustness of the model, several diagnostic procedures were conducted. Stationarity was assessed using Dickey-Fuller tests, and autocorrelation patterns were evaluated through visual inspection of the autocorrelation and partial autocorrelation plots, supplemented by the Durbin-Watson test. Sensitivity analyses were performed using alternative lag specifications to verify the stability of model estimates under different autocorrelation assumptions.

The analytic dataset included all laboratory-confirmed PCR COVID-19 cases reported in Duarte Province and in all other provinces of the DR. Suspected cases lacking PCR confirmation were excluded, as were days with missing data, although no missing observations occurred during the study period. The comparison group consisted of all provinces except Duarte, which did not implement the targeted intervention during the early outbreak.

All statistical analyses were performed using STATA version 15 (StataCorp LLC, College Station, Texas), and the code and commands used for model implementation are available upon request.

## Results

Lockdown policy

The initial number of new confirmed cases in Duarte Province before implementation of the lockdown policy was estimated at 0.45 and had a daily growth of 0.88 before the implementation of the policy, which was statistically significant (p < 0.001). The slope of changes in new confirmed cases following implementation of the social distancing policy decreased by 1.63, which was statistically significant (p < 0.001) (Table 1).

**Table 1:** Coefficients of the segmented regression model for lockdown policy

Parameters	Coefficients	Newey-West Standard Errors	t	p	95% CI	
Intervention	46.064	10.077	4.57	0.000	25.843	66.284
Time	0.884	0.253	3.48	0.001	0.375	1.393
Time after	-1.630	0.333	-4.89	0.000	-2.29851	-0.962
Constant	-0.474	2.379	-0.2	0.843	-5.2484	4.301
Number of observations	56					
Maximum lag	1					
F (3, 52)	10.93					
Probability > F	0.000					

On the other hand, the initial number of new confirmed cases in all provinces except Duarte before implementation of the lockdown policy was estimated at 29 and had a daily growth of 6 before the implementation of the policy, which was statistically significant (p < 0.001). The slope of changes in new confirmed cases following implementation of the social distancing policy decreased by 0.88, which was not statistically significant (p = 0.723) (Table 2).

**Table 2:** Coefficients of the segmented regression model for the lockdown policy except for Duarte

Parameters	Coefficients	Newey-West Standard Errors	t	p	95% CI	
Intervention	5.782	88.031	0.07	0.948	-170.865	182.430
Time	5.893	0.928	6.35	0	4.030	7.756
Time after	-0.813	2.279	-0.36	0.723	-5.386	3.759
Constant	28.978	13.799	2.1	0.041	1.287	56.669
Number of observations	56					
Maximum lag	1					
F (3, 52)	35.73					
Probability > F	0.000					

## Discussion

Public value refers to the value created by the government through services, laws, regulations, and other actions. It is produced by public managers successfully navigating a strategic triangle encompassing the following: producing valued outcomes, in this case clearly defined by international epidemiological metrics; operating within the constraints of available resources and capability; and working in an authorizing environment of formal and informal jurisdiction, legal frameworks, and mandate. This model attempts to articulate a clear and compelling idea of the public value that the agency exists to produce, develop a set of measures to record the agency’s performance in producing that public value, invite and embrace external accountability for defining and creating that value, and create management systems that distribute internal accountability for public value creation across managers and employees so that they will feel motivated to perform in the short run and to innovate and learn over the long run [19].

From this point of view, there are two sources of public value: those values that result from improving the government itself as an asset to society, and values that result from the delivery of specific benefits directly to persons or groups.

Moore acknowledges that among the problems left unresolved by Creating Public Value, “the most obvious issue was the difficulty of giving a clear, objective definition of what constitutes public value.” In our case, the roadmap to value was clearly defined by international standards: flatten the curve, represented by positivity rate; increase hospital capacity and availability; and reduce mortality, represented by case fatality ratio and gross mortality.

In a public health crisis, public value can be instituted as an organizing principle in a public sector organization, providing a focus through which collective response agencies remain aligned on the same goals and principles. In this case, the goals were already determined by international organizations: flatten the curve, increase testing capacity, enhance hospital capacity, and ultimately reduce positivity rate and case fatality rate, all through articulating public and private services in terms of efficiency [20].

In the case of #PlanDuarte all these elements were executed successfully with a strong public-private partnership. The measurement of outcomes and performance was directly addressed by standard COVID-19 indicators as reflected in this article. By controlling epidemiological spread in Duarte as a hotspot, the pandemic was able to be controlled and begin to de-escalate, opening the economy in various phases, decongesting hospitals, and allowing for economic growth. An important element in the success of this model was the empowerment of the local community, where everyone had buy-in, understanding that protecting their community was the correct approach. Families were empowered to provide solutions with home health and telemedicine options, local business leaders understood that by creating a healthier community, their business would thrive, and the private and public healthcare systems understood the value of scalability and integrating technologies to offer combined solutions. By creating a community empowerment culture, the long-term repercussions of this model can be seen one year later, as Duarte maintains the lowest provincial COVID-19 indicators.

The results indicate that the intervention implemented in Duarte Province was associated with a clear deceleration in COVID-19 case growth, closely aligning with the rollout of the Public Value Crisis Model. This improvement appears to have emerged from the combined effect of several coordinated measures, including strict movement restrictions, expansion of testing capacity, reinforcement of hospital infrastructure, and sustained community engagement. Together, these actions produced measurable epidemiological gains during a critical period of the outbreak.

The analysis does not permit definitive causal claims. Limited testing capacity in the early phase of the epidemic likely resulted in underestimation of actual case numbers, introducing potential measurement bias. The presence of multiple overlapping interventions, ranging from lockdown policies to home-visit medical units and expanded isolation capacity, further complicates any attempt to attribute observed improvements to a single element of the response. In addition, the unique political, social, and logistical conditions of Duarte Province limit the generalizability of these findings to other regions or countries.

Nonetheless, #PlanDuarte offers an instructive example of how a public value-oriented governance model, anchored in interagency coordination and empowered local participation, can strengthen crisis response in a middle-income setting. The experience underscores the importance of integrating epidemiological intelligence, operational capacity, and community collaboration in managing fast-moving public health emergencies.

Several limitations must be acknowledged. Early under-testing produced unavoidable measurement bias and restricted the precision of early trend estimates. The concurrent implementation of multiple policy actions limits the ability to isolate the contribution of any single intervention. Potential confounders, such as changes in population mobility, variation in community compliance, and broader national-level policies, were not fully controllable within the design. Data quality challenges, common in the early months of the pandemic, may also have influenced reported counts. Furthermore, the ITSA approach identifies associations rather than establishing causation, and the external validity of the findings is constrained by the specific contextual conditions of Duarte Province.

Our findings show that implementation of the Public Value Crisis Model in Duarte Province was associated with significant improvements in key epidemiological indicators, including reduced daily new cases and improved testing and hospital capacity. These improvements align with the model’s core components, which include coordinated public-private partnerships, community engagement, and intelligence-driven operational management.

However, consistent with ITSA limitations, these associations cannot be interpreted as definitive causal proof. The effectiveness of #PlanDuarte is also influenced by contextual factors such as political cooperation, local leadership, and community compliance. Furthermore, generalizability beyond the DR remains limited.

## Conclusions

Although responsibility for the pandemic cannot be placed solely on the shoulders of any single person, group, or institution, one of the great tragedies of the COVID-19 pandemic is that some of the worst outcomes could have been avoided had our predicament been acknowledged and acted upon at the appropriate time. Public value models are demonstrating the importance of community mobilization around local, regional, and central leaderships. Collective action is key to stop the spread of the pathogen. Closing borders, testing and tracing, bans on gatherings, school closures, and stay-at-home policies, such as Quedate en Casa, along with political turmoil, may provoke resentment and even resistance; therefore, leadership remains a quintessential value to sustain the model of intervention.

The Public Value Crisis Model applied through #PlanDuarte was associated with improved epidemiological outcomes in Duarte Province during the early COVID-19 outbreak. This localized intervention demonstrates how coordinated governance, public value principles, and community engagement can strengthen crisis response. While the findings reflect temporal associations, they provide a framework for data-informed, community-anchored policy design in low- and middle-income settings.
